# Repercussions of the COVID-19 pandemic on Nursing training: A Scoping Review*

**DOI:** 10.1590/1518-8345.6414.3912

**Published:** 2023-05-12

**Authors:** Anália Andréia de Araújo Nascimento, Sara Eloise Argimiro Ribeiro, Anne Caroline Lisboa Marinho, Valéria Dantas de Azevedo, Marina Eduarda Mendonça Moreira, Isabelle Campos de Azevedo

**Affiliations:** 1 Universidade Federal do Rio Grande do Norte, Natal, RN, Brasil.; 2 Becaria del Conselho Nacional de Desenvolvimento Científico e Tecnológico (CNPq), Brasil.; 3 Universidade Federal do Rio Grande do Norte, Departamento de Enfermagem, Natal, RN, Brasil.; 4 Becaria de la Coordenação de Aperfeiçoamento de Pessoal de Nível Superior (CAPES), Brasil.

**Keywords:** Nursing, Education, Nursing, Teaching, Education, Distance, Pandemics, COVID-19, Enfermería, Educación en Enfermería, Enseñanza, Educación a Distancia, Pandemias, COVID-19, Enfermagem, Educação em Enfermagem, Ensino, Educação à Distância, Pandemias, Infecção por Vírus COVID-19

## Abstract

**Objective:**

to map the knowledge produced about the repercussions imposed by the COVID-19 pandemic on Nursing training.

**Method:**

this is a Scoping Review, guided by the recommendations set forth in the Joanna Briggs Institute Reviewer’s Manual and carried out in 15 electronic databases and theses and dissertations repositories. The protocol was registered at the Open Science Framework. The data were analyzed and synthesized into two pre-established analysis categories: positive and negative repercussions; and descriptive statistics.

**Results:**

33 publications identified, the most cited positive aspects were the development of new teaching strategies adapted to the virtual environment and the training of future professionals in clinical practice in the context of a health crisis. The negative repercussions are related to psychological issues such as increase in the cases of anxiety, stress and loneliness among the students.

**Conclusion:**

the diverse evidence suggests that remote teaching was a timely emergency way out for the continuity of academic training; however, this educational modality presented positive and negative aspects that need to be rethought for a better systematization of teaching-learning in other contexts that resemble the COVID-19 pandemic.

## Abstract (es)

**Objetivo:**

mapear el conocimiento producido sobre las repercusiones de la pandemia de COVID-19 en la formación en enfermería.

**Método:**

se trata de una *scoping review*, guiada por las recomendaciones del *Joanna Briggs Institute Reviewer’s Manual* realizada en 15 bases de datos electrónicas y repositorios de tesis y disertaciones. El protocolo de esta revisión fue registrado en Open Science Framework. Los datos fueron analizados y sintetizados en dos categorías de análisis establecidas: aspectos positivos y negativos y estadística descriptiva.

**Resultados:**

se identificaron 33 publicaciones, los aspectos positivos más citados fueron el desarrollo de nuevas estrategias de enseñanza adaptadas al entorno virtual y la formación de futuros profesionales en la práctica clínica en el contexto de una crisis sanitaria. Los aspectos negativos se relacionan con cuestiones psicológicas, como el aumento de casos de ansiedad, estrés y soledad en los estudiantes.

**Conclusión:**

la evidencia sugiere que la enseñanza a distancia fue una solución de emergencia oportuna para continuar con la formación académica, sin embargo, esta modalidad educativa presentó aspectos positivos y negativos que deben ser repensados para que haya una mejor sistematización de la enseñanza-aprendizaje en otros contextos similares al de la pandemia de COVID-19.

## Abstract (pt)

**Objetivo:**

mapear o conhecimento produzido sobre as repercussões da pandemia COVID-19 na formação em Enfermagem.

**Método:**

trata-se de uma *scoping review*, guiada pelas recomendações da *Joanna Briggs Institute Reviewer’s Manual* realizada em 15 bases de dados eletrônicas e repositórios de teses e dissertações. O protocolo desta revisão foi registrado em *Open Science Framework*. Os dados foram analisados e sintetizados em duas categorias de análise estabelecidas: aspectos positivos e negativos e estatística descritiva.

**Resultados:**

33 publicações identificadas, os aspectos positivos mais citados foram o desenvolvimento de novas estratégias de ensino adaptadas ao meio virtual e a capacitação dos futuros profissionais na prática clínica do contexto de crise sanitária. Os aspectos negativos estão relacionados às questões psicológicas, como aumento de casos de ansiedade, estresse e solidão entre os estudantes.

**Conclusão:**

as evidências sugerem que o ensino remoto foi uma saída emergencial oportuna para a continuidade da formação acadêmica, contudo, esta modalidade educacional apresentou aspectos positivos e negativos que precisam ser repensados para uma melhor sistematização do ensino-aprendizagem em outros contextos que se assemelhem ao da pandemia da COVID-19.

**Highlights:**

**(1)** This review mapped the diverse scientific evidence about the repercussions imposed by the pandemic on Nursing training.

**(2)** It also highlighted the positive and negative repercussions of the pandemic on Nursing training.

**(3)** It evidenced the importance of Nursing training in the pandemic context.

**(4)** Finally, it pointed out teachers’ training for remote teaching in Nursing.

## Introduction

The COVID-19 pandemic context imposed changes in society and challenged public authorities, which began to adopt biopolitical control devices to regulate people’s lives, encouraging social distancing and home office^(1)^. Among the changes that affected education there is emergency remote teaching, guided by the Ministry of Education as a temporary substitute model for face-to-face classes, according to Ordinance No. 343, of March 17^th^, 2020, in order to reduce the contagion caused by the virus and continue the students’ training process.

The demands inherent to this type of teaching are countless, such as access to energy, Internet, digital technologies, software programs and learning materials^(2)-(3)^. It is important to note that, according to data from the Brazilian Institute of Geography and Statistics (*Instituto Brasileiro de Geografia e Estatística*, IBGE), one out of four Brazilians has no Internet access, i.e., 46 million people^(4)^.

Virus contagion prevention strategies in education, supported by the use of information and communication technologies, constituted a way to minimize the losses caused in this field by social isolation, providing the possibility for students to have access to a teaching-learning process^(3)^.

A number of research studies carried out with Nursing students at the technical level point out that several factors have directly affected the students’ quality of life and, therefore, their learning; variables such as economic and social vulnerability, changes in routine, social distancing, risk situations in families, household chores, non-differentiation between the study environment and the home/family environment and multiple remote and face-to-face functions, in addition to impaired mental health, are factors that end up related to low attendance to classes and a greater deficit in learning^(5)-(7)^.

Other evidence points out that most higher education Nursing students belong to families of intermediate or low social levels^(8)^; therefore, it can be inferred that some students may not have access to the Internet and/or stable computers for use during remote teaching, nor a support network to maintain the current activities, as they need to work to pay for part of the household/family/personal expenses^(6)-(8)^.

The study is also justified by the need to discover the possible repercussions on Nursing students both at the technical and higher level, related to the issues already mentioned, as well as the concern with the difficulty of their own training and the possibility of finding a job or enrolling in a graduate course, by postponing^(9)^ or advancing degrees^(10)^ due to the lack of professionals in view of the high demand for health services, which can harm consolidation of theoretical knowledge, development of technical skills and decision-making, fundamental competencies for future professionals^(10)^.

From a training point of view, emergency remote teaching does not fully meet the requirements of Nursing education, considering that, in addition to theoretical content, the students need to practice the techniques and procedures in health services in order to check skills and competencies necessary for their graduation profile. Such experiences should preferably take place through direct interaction with patients, handling equipment and materials intended for health care, and interprofessional relationships^(10)-(11)^.

Ensuring the effective implementation of the teaching-learning process in Brazilian Nursing courses in the pandemic scenario emerges as a challenge. In the meantime, it is understood that there is an urgent need to know the positive and negative repercussions impacts that emergency remote teaching has exerted on the training and professionalization of new nurses, as a way of carrying out a situational diagnosis of the strengths and weaknesses of this type of teaching and to have a starting point to take action on what needs to be rescued or worked on in greater depth to equip students to strengthen their graduation profile. To such end, the objective was to map the knowledge produced about the repercussions imposed by the COVID-19 pandemic on Nursing training.

## Method

### Type of study

This study is a scoping review guided by the recommendations set forth in the Joanna Briggs Institute Reviewer’s Manual (JBI)^(12)^ and developed in five stages, namely: Formulation of the research question; Identification of the relevant studies; Selection of the studies; Data extraction and analysis; and Synthesis and reporting. The research protocol was registered in the Open Science Framework (OSF) (https://osf.io/zyq84/), and follows the Preferred Reporting Items for Systematic reviews and Meta-Analyses extension for Scoping Reviews (PRISMA-ScR)^(13)^.

This type of study aims at investigating the main evidence for a specific knowledge area by researching available scientific productions and possible gaps on the topic addressed. And, to ensure that there are no studies with the same theme registered in OSF or published, a broad search was carried out on the platform and in databases to identify protocols or reviews with a similar theme. Based on such diagnosis, the stages to consolidate this review were conducted.

### Setting

This review was developed in the following thesis and dissertation databases and repositories: National Library of Medicine (PubMed); SCOPUS; Web of Science (WoS); Science Direct, *Literatura Latino-Americana e do Caribe em Ciências da Saúde* (LILACS), The National Library of Australia’s Trobe (TROVE), Academic Archive Online (DIVA), CAPES, Education Resources Information Center (ERIC), DART-Europe E-Theses Portal, Electronic Theses Online Service (EThOS), *Repositório Científico de Acesso Aberto de Portugal* (RCAAP), National ETD Portal, Theses Canada, and *Teses e Dissertações da América Latina*.

### Period

The data searches were performed between March and April 2022.

### Selection criteria

Studies published in full and publications that met the research objective and guiding question were included, available in full through remote access from the Federated Academic Community (*Comunidade Acadêmica Federada*, CAFe), on the Journals Portal of the Coordination for Improvement of Higher Education Personnel (*Coordenação de Aperfeiçoamento de Pessoal de Nível Superior*, CAPES) belonging to the Ministry of Education (MEC). The exclusion criteria were as follows: studies in the editorial format, letters to the editor and opinion articles. Duplicate documents were considered only once. It is important to note that no time frame was applied.

### Data collection

The first stage is related to formulating the research question, guided by the PCC strategy (P=Population: Nursing; C=Concept: Education in Nursing; C=Context: COVID-19 pandemic). Therefore, the guiding question that was prepared to achieve the objective of this study was as follows: Which is the available scientific evidence about the repercussions of the COVID-19 pandemic in Nursing training?

In order to develop the second stage, four different search strategies were devised, as described in Figure1. The descriptors and synonyms in the English and Portuguese languages were defined by means of the Medical Subject Headings (MeSH) and of the Descriptors in Health Sciences (*Descritores de Ciência da Saúde*, DeCS), respectively. In addition to that, the AND and OR Boolean operators were used.

**Figure 1 -:** Search expressions used in the databases. Natal, RN, Brazil, 2022

Databases	Search terms
LILACS	#1– *enfermagem AND educação em enfermagem AND pandemias* #2– *enfermagem AND educação à distância AND pandemias*
Science Direct in the CAPES Thesis and Dissertation Catalog and in *Teses e Dissertações da América Latina*, in DIVA, in ERIC, in TROVE, in DART, in ETD, and in SCOPUS	#1– *nursing AND nursing education AND pandemics* #2– *nursing AND distance education AND pandemics*
SCOPUS	#1– ALL (*nursing*) OR (*nursing, students* OR *nursing, practice* OR *mentors*) AND (*education nursing*) OR (*professional education* OR *distance education* OR *nursing education research*) AND (*pandemics*) OR (SARS-CoV-2 OR COVID-19 *virus infection*). #2 –ALL (*nursing*) OR (*nursing, students* OR *nursing, practice* OR *mentors*) AND (*distance education*) OR (*education nursing* OR *professional education* OR *nursing education research*) AND (*pandemics*) OR (SARS- CoV-2 OR COVID-19 *virus infection*)
Search strategy for the other databases	#1– *nursing* (*nursing, students* OR *nursing, practice* OR *mentors*) AND *education nursing* (*professional education* OR *distance education* OR *nursing education research*) AND *pandemics* (SARS-CoV-2 OR COVID-19 *virus infection*). #2– *nursing* (*nursing, students* OR *nursing, practice* OR *mentors*) AND *distance education* (*education nursing* OR *professional education* OR *nursing education research*) AND *pandemics* (SARS-CoV-2 OR COVID-19 *virus* *infection*)

The third stage of the study consists in selecting and evaluating the studies; all the bibliographical references retrieved were the result of searches in the literature, initially pre-analyzed from reading the titles and abstracts, at the same time that the eligibility criteria were applied.

### Data treatment and analysis

The fourth stage consisted in data extraction and analysis, performed independently by two researchers who, in case of disagreement, discussed the issue to reach consensus. In case of doubts or disagreements, a third reviewer specialized in the study object area issued his/her opinion.

The studies were organized in a *Microsoft Excel* ^®^ spreadsheet, specifically designed to extract the following variables: author, country where the study was carried out, year of publication, type of study, repercussions of the pandemic on Nursing education and level of evidence. To perform the fifth stage, the aforementioned tool allowed for the synthesis, data interpretation and a basic numerical analysis of the extension, nature and distribution of the studies selected to comprise the final sample based on the recommendations of a study^(14)^. The level of evidence of the studies was classified according to the JBI^(12)^. The data were analyzed by using simple statistics in a descriptive way, by presenting the absolute and relative frequencies, and they were presented in the form of figures and charts.

### Ethical aspects

For being a research study conducted with secondary data in the public domain and available in the literature, no ethical appreciation was necessary. However, it is worth noting that copyright was respected with due citation and referencing of the studies.

## Results

**Figure 2 -:**
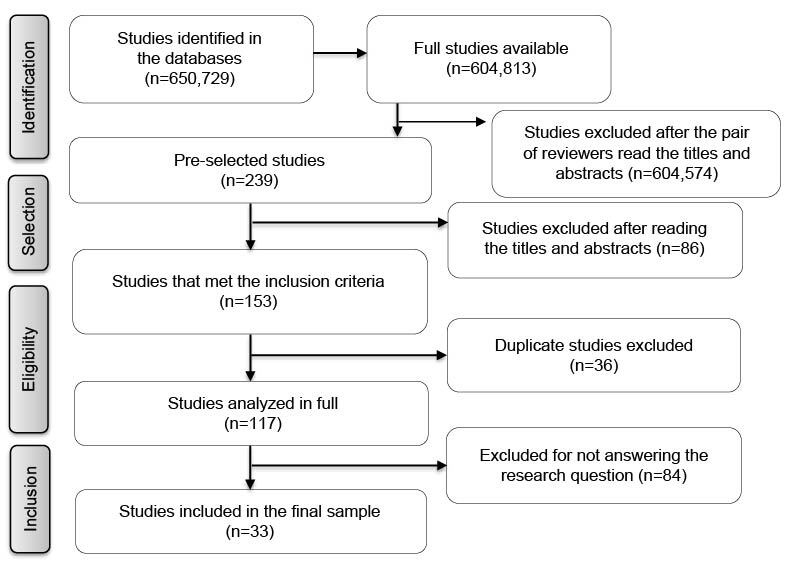
Flowchart corresponding to the selection process (n=33). Natal, RN, Brazil, 2022

Initially, 650,729 studies were identified in the dissertation and thesis databases and repositories listed; however, only 604,813 were available in full for the analysis. After reading the titles and abstracts, the pair of reviewers excluded 604,574 documents; therefore, only 239 articles were selected for full-reading. After the selection process, described in Figure2, 33 articles comprised the final sample.

As for the year, it was verified that, in 2021, 20 (60.60%) studies were published that dealt with the impacts of the COVID-19 pandemic on Nursing education, followed by 2022 (8; 24.24%) and 2020 (5; 15.15%). In relation to language, seven (21.21%) are written in Portuguese and 26 (78.78%) in English.

Among the countries in which the research studies were developed, Brazil stands out with 10 articles (30.30%), the United States of America (USA) with four (12.12%), two of them (6.06%) multicenter studies, Spain, South Korea, Turkey, Iran and China also with two (6.06%) each, and Israel, Ireland, United Kingdom, Nepal, Australia, Belgium and Indonesia were responsible for one (3.03%) publication per country.

In order to systematize the results in Figures3 and 4, the final sample was described in studies that evidenced the positive and negative repercussions of the COVID-19 pandemic on Nursing training.

Figure3 shows the characterization of the studies selected in the scoping review according to country, type of study and positive repercussions of the pandemic on Nursing training.

**Figure 3 -:** Characterization of the studies regarding authors and year of publication, country where the research was carried out, type of study, synthesis of the positive impacts of the pandemic on Nursing education and level of evidence (n=33). Natal, RN, Brazil, 2022

ID*	Reference	Country	Type of study	Positive repercussions of the pandemic on Nursing training	Level of Evidence
A1†	Warshawsk S.^(15)^	Israel	Cross-sectional	Resilience and social support from family members and friends.	IV‡
A2†	Saab MM, Hegarty J, Murphy D, Landers M.^(16)^	Ireland	Qualitative and descriptive	New, inclusive and participatory learning environment.	IV‡
A3†	Kalanlar B.^(17)^	Turkey	Cross-sectional	The participants believe that the good results of the Nursing program were achieved by means of education at a distance.	IV‡
A4†	Shorey S, Ang E, Baridwan NsS, Bonito SR, Dones LBP, Flores JLA, et al. ^(18)^	Multicenter	Qualitative and descriptive	The participants were satisfied because it was more comfortable and for the possibility of studying at their homes with the new teaching modalities; good social well-being levels.	IV‡
A5†	Kunaviktikul W, Ang E, Baridwan NS, Bernal AB, Dones LBP, Flores JL, et al.^(19)^	Multicenter	Qualitative and descriptive	The students rated on-line education as appropriate, good and valuable; new sources of resilience; reaffirmation of the choice of Nursing as a profession; convenience of remote learning; reassessment of teaching pedagogies.	IV‡
A6†	Canet-Vélez O, Botigué T, Lavedán Santamaría A, Masot O, Cemeli T, Roca J.^(20)^	Spain	Qualitative and descriptive	The students were able to notice the gaps resulting from the training process and that were necessary to face the crisis; valuing nurses’ skills and competencies.	IV‡
A7†	Godbold R, Whiting L, Adams C, Naidu Y, Pattison N. ^(21)^	United Kingdom	Qualitative and descriptive	Possibility of a clinical experience during the pandemic crisis; development of autonomy and self-confidence by the students; learning new skills and updates.	IV‡
A8†	Zaragoza-García I, Ortuño- Soriano I, Posada-Moreno P, Sánchez-Gómez R, Raurell- Torredà M.^(22)^	Spain	Quasi-experimental	Development of a virtual reality environment to assist in the students’ training process; using this technology promoted an improvement in the acquisition of knowledge and skills by means of a training program.	III^§^
A9†	Park J, Seo M.^(23)^	South Korea	Mixed and sequential	The online classes allowed the students to choose the learning time and place; they also allowed repeating the classes; the students were forced to self-regulate their learning.	IV‡
A10†	Nodine PM, Arbet J, Jenkins PA, Rosenthal L, Carrington S, Purcell SK, et al.^(24)^	United States of America	Cohort	More resilience; possibility of attending classes from the students’ homes.	III^§^
A11†	Scorsolini-Comin F, De Melo LP, Rossato L, Gaia RDSP.^(25)^	Brazil	Reflection	The pandemic as an element to reheat the debate on remote Nursing training, critically and based on evidence.	IV‡
A12†	Bastos MC, Canavarro DA, Campos LM, Schulz RS, Santos JB, Santos CF.^(26)^	Brazil	Experience report	Teachers’ training on the use of online platforms; approach by the course coordination to the students; elaboration of new methodologies adapted to the context.	IV‡
A13†	Rodrigues PS, Marin MJS, Souza AP, Grandin GM, Almeida KRV, Oliveira CSR.^(27)^	Brazil	Qualitative and descriptive	Need for integration among peers and good group dynamics.	IV‡
A14†	Silva CB, Trindade LL, Kolhs M, Barimacker SV, Schacht L, Bordignon M.^(6)^	Brazil	Theoretical- reflective	Development of educational methodologies adapted to remote teaching; autonomy of remote teaching; ease of study in various spaces; need to promote the elaboration of efficient and inclusive teaching strategies; need to present topics that returned to the discussion agenda, such as mental health and professionals’ safety.	IV‡
A15†	Soccol KLS, Santos NO, Marchiori MRCT.^(28)^	Brazil	Reflection	It enabled students to develop competencies and skills in a crisis context; strengthening of learning in the face of the new and the information that arises; the pandemic scenario exposed the extent to which Nursing is undervalued.	IV‡
A16†	Li W, Gillies R, He M, Wu C, Liu S, Gong Z, et al.^(29)^	China	Qualitative and descriptive	The teachers were satisfied with the online education experiences; possibility for students from outside China to complete the remote course; the family environment as support to overcome negative emotions; greater commitment and self-discipline to the learning process by the students.	IV‡
A17†	Emory J, Kippenbrock T, Buron B.^(30)^	United States of America	Cross-sectional	Exposure to deficit in training on safety and adequate use of Personal Protective Equipment.	IV‡
A18†	Riegel F, Martini JG, Bresolin P, Mohallem AGC, Nes AAG. ^(31)^	Brazil	Theoretical- reflective	It sparked the need for an innovative approach to the development of curricula and new learning projects; development of new technologies to add quality to the teaching-learning process; development of different teaching methods and models.	IV‡
A19†	Baixinho CL, Ferreira OR.^(32)^	Brazil	Qualitative and descriptive	Need to explore new pedagogical strategies and to evaluate their effectiveness; the students’ participation in the clinical practice reinforces care in times of crisis.	IV‡
A20†	Salmani N, Bagheri I, Dadgari A.^(33)^	Iran	Qualitative and descriptive	Remote teaching allowed self-centered flexible learning and reducing the concerns about in-person learning.	IV‡
A21†	Tolyat M, Abolfazl Vagharseyyedin S, Nakhaei M. ^(34)^	Iran	Qualitative and descriptive	Development of new teaching methods adapted to remote teaching; shift from teacher-centered to student- centered education; the media resources highlighted nurses’ role in treating diseases; fewer students in the training rooms.	IV‡
A22†	Thapa P, Bhandari SL, Pathak S.(35)	Nepal	Cross-sectional	Remote teaching enables home-based study with reduced costs; development of new teaching methods adapted to the remote modality; greater learning ease.	IV‡
A23†	Lei T, Yu X, Zou M, Wang P, Yuan RH.^(36)^	China	Quasi-experimental	Remote teaching can strengthen information; providing an additional demonstration of concepts; encouraging active learning and intuitive animation; teaching through videos.	III^§^
A24†	Wynter K, Holton S, Considine J.(37)	Australia	Qualitative and descriptive	Development of new online learning methods; flexible education; acquisition of new skills and knowledge; increased productivity by working remotely and participating in virtual meetings; appreciation of Nursing as an important profession in the fight against the virus.	IV‡
A25†	Susmarini D, Sumarwati M, Handayani F, Iskandar A.^(38)^	Indonesia	Qualitative and descriptive	Emergence of a sense of pride for being part of the Nursing workforce; development of skills and mastery of the technology; more effective communication.	IV‡
A26†	Michel A, Ryan N, Mattheus D, Knopf A, Abuelezam NA, Stamp K, et al.^(39)^	United States of America	Cross-sectional	Flexibility and openness to unknown situations; the pandemic showed that Nursing schools need to devise suitable plans for disasters.	IV‡
A27†	Araújo ARL, Sousa LMC, Carvalho RBS, Oliveira ADS, Amorim FCM, Sousa KHJF, et al.^(40)^	Brazil	Cross-sectional	Need to develop new teaching methodologies adapted to remote teaching; possibility of home-based work; development of technological skills; better communication; it enhanced teachers’ professional development; it highlighted the need to improve relationships with students, colleagues and coordination.	IV‡
A28†	Terzi B, Azizoğlu F, Özhan F. ^(41)^	Turkey	Cross-sectional	Development of suitable educational methods in the online environment; current classes; it encouraged the student-professor interaction; improved informatics skills.	IV‡
A29†	Silva CM, Toriyama ATM, Claro HG, Borghi CA, Castro TR, Salvador PICA^(42)^	Brazil	Experience report	Development of a new methodology adapted to remote teaching that minimizes the impact of interruption of classes; the students’ possibility to attend recorded classes at a suitable moment.	IV‡
A30†	Duprez V, Vermote B, Van Hecke A, Verhaeghe R, Vansteenkiste M, Malfait S.^(43)^	Belgium	Cross-sectional	Greater commitment to the choice of studying Nursing by final year students; greater clarity about the value of Nursing.	IV‡
A31†	Jones K, Polyakova-Norwood V, Raynor P, Tavakoli A.^(44)^	United States of America	Mixed methods	Behavioral change in the teachers, becoming more understanding in terms of deadlines and open communication.	IV‡
A32†	Kim S, Jeong SH, Kim HS, Jeong YJ.^(45)^	South Korea	Cross-sectional	Encouraging continuous self-directed learning; shift from an instructor-centered to a student-centered teaching model; greater communication between professors and students; need for instructor feedback for better learning; better academic performance; development of new educational methods that increase students’ engagement.	IV‡
A33†	Prata JA, Mello AS, Silva FVC, Faria MGA.^(46)^	Brazil	Experience report	Need to develop educational methods adapted to online teaching; creation of online, didactic and updated courses.	IV‡

* ID = Identification;

† A = Article;

‡ IV = Evidence from experts’ opinion, descriptive studies, case reports or experts’ committee reports^(12)^;

^§^ III = Evidence from analytical studies, controlled clinical trials without randomization, cohort, case-control, quasi-experimental studies^(12)^

Figure 4 presents the characterization of the studies selected in the scoping review according to country, type of study and negative repercussions of the pandemic on Nursing training.

**Figure 4 -:** Characterization of the studies regarding authors and year of publication, country where the research was carried out, type of study, synthesis of the negative impacts of the pandemic on Nursing education and level of evidence (n=33). Natal, RN, Brazil, 2022

ID*	Reference	Country	Type of study	Negative repercussions of the pandemic on Nursing training	Level of Evidence
A1†	Warshawsk S.^(15)^	Israel	Cross-sectional	Difficulties perceived by the students, such as overload, difficulty with online learning, lack of academic support, lack of social interaction.	IV‡
A2†	Saab MM, Hegarty J, Murphy D, Landers M.^(16)^	Ireland	Qualitative and descriptive	High cost, threats to human connections, potential side effects and lack of interest.	IV‡
A3†	Kalanlar B.^(17)^	Turkey	Cross-sectional	Internet-related difficulties, such as connection failures or poor-quality connections.	IV‡
A4†	Shorey S, Ang E, Baridwan NsS, Bonito SR, Dones LBP, Flores JLA, et al.^(18)^	Multicenter	Qualitative and descriptive	The students felt that their training was impaired; high stress levels.	IV‡
A5†	Kunaviktikul W, Ang E, Baridwan NS, Bernal AB, Dones LBP, Flores JL, et al.^(19)^	Multicenter	Qualitative and descriptive	Increased feelings of loneliness, depression, sadness, boredom, fear, anxiety, stress, worry and vulnerability among the students; unpreparedness to return to their Nursing duties after their absence from the clinical setting; loss of boundaries between work and home; difficulties carrying out group work due to little interaction; technical problems during learning that greatly affected both teaching and learning.	IV‡
A6†	Canet-Vélez O, Botigué T, Lavedán Santamaría A, Masot O, Cemeli T, Roca J.^(20)^	Spain	Qualitative and descriptive	Only a minority of students felt prepared to face the COVID-19§ crisis; overload, wear out, exhaustion and high stress levels; feelings of fear, worry and tense situations of nervousness.	IV‡
A7†	Godbold R, Whiting L, Adams C, Naidu Y, Pattison N. ^(21)^	United Kingdom	Qualitative and descriptive	Need to distance from the family to comply with the clinical practices; due to staff shortages, the students worked with people relocated from other areas; the emotional issues raised by the social distancing restrictions that did not allow using touch and physical comfort; the difficulties of studying at home and simultaneously taking care of the family; physical effects from the use of Personal Protective Equipment, such as sore hands and marks on the face.	IV‡
A8†	Zaragoza-García I, Ortuño- Soriano I, Posada-Moreno P, Sánchez-Gómez R, Raurell- Torredà M.^(22)^	Spain	Quasi-experimental	The platform intensifies social distancing.	III^||^
A9†	Park J, Seo M.^(23)^	South Korea	Mixed and sequential	Technical difficulties such as disconnections; decreased concentration and motivation; fatigue due to the long period using digital devices; changes in sleep patterns; increased depression, anxiety and fear of COVID-19^§^; classes had to be short so that concentration and motivation to learn did not diminish; absence of face-to-face clinical practice during the pandemic; sudden pauses in the in-hospital clinical practices; decreased opportunities during the practices; need to find suitable places to study because the school was closed.	IV‡
A10†	Nodine PM, Arbet J, Jenkins PA, Rosenthal L, Carrington S, Purcell SK, et al.^(24)^	United States of America	Cohort	Increased stress levels in all the subscales evaluated, including course didactics, papers, family, clinical turnovers, finance, illness in family members and/or friends.	III^||^
A11†	Scorsolini-Comin F, De Melo LP, Rossato L, Gaia RDSP.^(25)^	Brazil	Reflection	High evasion rates among the students in this teaching modality.	IV‡
A12†	Bastos MC, Canavarro DA, Campos LM, Schulz RS, Santos JB, Santos CF.^(26)^	Brazil	Experience report	Teacher-student distancing; low adherence to remote teaching among the students due to distractions in home-based learning; increased workload in teachers and students.	IV‡
A13†	Rodrigues PS, Marin MJS, Souza AP, Grandin GM, Almeida KRV, Oliveira CSR.^(27)^	Brazil	Qualitative and descriptive	Technological difficulties; emergence of feelings such as anxiety, anguish, discouragement, stress and tiredness; tiring and low-performance process; lower concentration; home environment as a difficulty for the learning process; lack of contact with classmates; lack of access to the library; lack of practices in laboratories.	IV‡
A14†	Silva CB, Trindade LL, Kolhs M, Barimacker SV, Schacht L, Bordignon M.^(6)^	Brazil	Theoretical- reflective	Need for better planning of virtual activities; difficulties accessing the Internet and information; increased stress and tiredness levels; work in an environment with other family members; feelings such as fear, anxiety and loneliness.	IV‡
A15†	Soccol KLS, Santos NO, Marchiori MRCT.^(28)^	Brazil	Reflection	Intensification of the feelings of insecurity and anxiety in the supervised internship.	IV‡
A16†	Li W, Gillies R, He M, Wu C, Liu S, Gong Z, et al.^(29)^	China	Qualitative and descriptive	The students were dissatisfied with the online education experiences; increase in future uncertainties; increased stress levels due to extended workdays and fatigue.	IV‡
A17†	Emory J, Kippenbrock T, Buron B.^(30)^	United States of America	Cross-sectional	Fear in the students about contaminating family members with the virus; high fear and anxiety levels; lack of quality learning; greater uncertainty about the future among the students; increase in cases of stress and mental exhaustion.	IV‡
A18†	Riegel F, Martini JG, Bresolin P, Mohallem AGC, Nes AAG. ^(31)^	Brazil	Theoretical- reflective	Access difficulties faced by the students during remote teaching; emergence of a feeling of loneliness; need for human and social interaction with professors and students.	IV‡
A19†	Baixinho CL, Ferreira OR.^(32)^	Brazil	Qualitative and descriptive	Impossibility of carrying out clinical teaching with COVID-19^§^ patients ; confinement for three months caused a setback in the ability to construct clinical reasoning; overload for services and professionals, who still assumed the role of clinical advisors; reduction in the clinical teaching time; increased fear of failure; fear of contact with others due to fear of infection; physical and psychological exhaustion; increased symptoms such as anxiety and poor sleep quality; reduction in terms of performance.	IV‡
A20†	Salmani N, Bagheri I, Dadgari A.^(33)^	Iran	Qualitative and descriptive	Changes in the way students interact with teachers; decreased interactions with peers; problems with educational files; shallow learning; family members’ perception of the student’s role; domestic affairs interfering with e-learning; cheating on tests and activities.	IV‡
A21†	Tolyat M, Abolfazl Vagharseyyedin S, Nakhaei M. ^(34)^	Iran	Qualitative and descriptive	Internet access difficulties; less teacher-student interaction; lengthy breaks between practices; decrease in practice time to avoid contagion; most of the students did not acquire the necessary clinical competence in Nursing education; reduction in the number of patients; greater insecurity among the students.	IV‡
A22†	Thapa P, Bhandari SL, Pathak S.(35)	Nepal	Cross-sectional	Internet problems and technical issues hindered remote teaching; lack of interaction with the patients.	IV‡
A23†	Lei T, Yu X, Zou M, Wang P, Yuan RH.^(36)^	China	Quasi-experimental	Absence of practices for the development of clinical skills.	III^||^
A24†	Wynter K, Holton S, Considine J.(37)	Australia	Qualitative and descriptive	Reduced social connection during learning; increase in teachers’ workload; uncertainties about how to carry out clinical practices; concern about whether the students would achieve the expected educational and clinical outcomes; onset of depression, anxiety, stress and burnout symptoms.	IV‡
A25†	Susmarini D, Sumarwati M, Handayani F, Iskandar A.^(38)^	Indonesia	Qualitative and descriptive	Emergence of feelings of fear and anxiety among students and family members; lack of clinical competence to deal with the crisis; rapid changes in the clinical practice sectors; financial burden for COVID-19^§^ testing during the practices.	IV‡
A26†	Michel A, Ryan N, Mattheus D, Knopf A, Abuelezam NA, Stamp K, et al.^(39)^	United States of America	Cross-sectional	Increased stress levels and feelings such as fear, loneliness and anxiety; concern about exposure to COVID-19§; connection difficulties; the difficulties of studying in a family environment; learning difficulties; increased workload; lack of faculty preparation for the virtual environment; lack of clinical experiences; feeling of being less engaged with education.	IV‡
A27†	Araújo ARL, Sousa LMC, Carvalho RBS, Oliveira ADS, Amorim FCM, Sousa KHJF, et al.^(40)^	Brazil	Cross-sectional	Work overload; change in domestic routine; little teacher-student interaction; emergence of feelings such as sadness, anguish, fear, anxiety, loneliness and stress; physical wear out, difficulty sleeping, poor diet; visual damage; obligation to quickly adapt to the digital environment.	IV‡
A28†	Terzi B, Azizoğlu F, Özhan F. ^(41)^	Turkey	Cross-sectional	Difficulty developing practical skills.	IV‡
A29†	Silva CM, Toriyama ATM, Claro HG, Borghi CA, Castro TR, Salvador PICA^(42)^	Brazil	Experience report	Difficulties adapting to the remote model among the faculty members and the students.	IV‡
A30†	Duprez V, Vermote B, Van Hecke A, Verhaeghe R, Vansteenkiste M, Malfait S.^(43)^	Belgium	Cross-sectional	The services failed to welcome the students due to the demands linked to COVID-19^§^.	IV‡
A31†	Jones K, Polyakova-Norwood V, Raynor P, Tavakoli A.^(44)^	United States of America	Mixed methods	Emergence of feelings such as loneliness, anxiety and stress; the students need more emotional support.	IV‡
A32†	Kim S, Jeong SH, Kim HS, Jeong YJ.^(45)^	South Korea	Cross-sectional	Lower concentration and attention levels in the online classes among the students; Internet access difficulties; high anxiety levels; difficulties to improve technological competence among the students.	IV‡
A33†	Prata JA, Mello AS, Silva FVC, Faria MGA.^(46)^	Brazil	Experience report	Internet access difficulties; preconceptions related to better leverage by the students.	IV‡

* ID = Identification;

† A = Article;

‡ IV = Evidence from experts’ opinion, descriptive studies, case reports or experts’ committee reports^(12)^;

§ COVID-19 = *Coronavirus Disease* 19;

|| III = Evidence from analytical studies, controlled clinical trials without randomization, cohort, case-control, quasi-experimental studies^(12)^

## Discussion

From the studies analyzed, it was possible to perceive that the COVID-19 pandemic exerted positive and negative repercussions on the training process of future nurses, related to the new routines imposed by social distancing. For a better understanding of the discussion of results, this research was organized into two topics, the first one dealing with positive repercussions and the second with negative impacts.

### Positive impacts of the pandemic on Nursing training

Almost half of the publications presented the need to develop new educational methods adapted to the virtual environment as a main positive repercussions of remote teaching or e-learning^(6),(16),(18),(22),(26),(31)-(32),(35)-(38),(40),(42),(45)-(46)^. Teachers needed to develop dynamic and engaging methodologies that increase engagement and value students’ concentration^(23)^. Some of the teaching strategies used were “synchronous” classes, that is, the professors deliver the class from their homes and the students are participating simultaneously; recorded video classes, which allowed repeating the contents as many times as necessary and, consequently, a better learning experience due to the multiple access instances^(23),(34),(36),(46)^ and activities through virtual simulations, which brought the students closer to the real practice^(16),(22)^.

Another advantage of this teaching model was the possibility of attending classes at home and at variable times in some institutions^(23)^. Studies carried out in Brazil^(40),(42)^ and China^(29)^ presented the opinions of students, mainly those who work, who valued the possibility of participating in classes at times adaptable to their routine and in other spaces, such as their own work environments^(29),(40),(42)^.

The opportunity for the students to develop competencies and skills in a crisis context was also cited in the research studies as a positive impact of the pandemic. Surveys carried out in Spain^(20)^, Brazil^(28),(32)^ and UK^(21)^ brought up the discussion that, before the pandemic, Nursing students did not feel prepared for a health crisis context, as the subject matter was approached theoretically, there was access to information, but there was no contact with the clinical practice and, given the context imposed by COVID-19, this skill could be better developed^(20),(32)^.

Although mentioned with less recurrence, the development of technological skills, both by professors and students, was also evidenced as an effect of the pandemic on Nursing teaching^(40)^; as well as encouraging self-directed learning, as it required greater concentration and focus on their own training process^(45)^; approximation of the teacher-student dyad, as communication needed to be improved and teachers became more flexible regarding direct contact through messaging apps^(44)^; the change in the traditional teaching method, from instructor-centered to student-centered, and greater flexibility in demands and deadlines for delivering activities was noticed^(46)^; and development of new technologies to add quality to the teaching-learning process, for example, the creation of virtual reality that brought theory closer to practice while maintaining distancing^(16)^.

In this way, it is noticed that the positive repercussions with regard to teaching in Nursing in the pandemic context had different implications for the students, mainly the development of new study skills and, for the teachers, expansion of knowledge and new skills for teaching with greater use of technologies and tools. In addition, it is a well-known fact that the pandemic context has made it possible to perceive and implement the importance of Nursing training in times of a health crisis, in addition to the fact that higher education institutions must have a contingency plan for the continuity of training in times like this and that it is also important to encourage professional development.

### Negative impacts of the pandemic on Nursing training

Psychological issues were the main negative repercussions of the pandemic on Nursing training cited in the studies. With social distancing and home confinement, many students and professors reported increased anxiety and stress levels, emergence of feelings such as loneliness, fear of infection, fear of contaminating family and friends, death, depression, sadness, vulnerability and a feeling of helplessness^(6),(15),(19)-(20),(22),(27)-(32),(37),(39)-(40),(44)^. Although some cases mentioned the fact of being in a familiar environment as a relief factor for these feelings^(15),(18)^, other studies show that teaching in a home environment hindered the learning process, as it was not an appropriate, silent setting that allowed concentration due to the presence of relatives and household chores that led to distractions for learning^(21),(23),(26)-(27),(39),(42)^.

Internet access difficulties were also raised as negative repercussions of the pandemic and remote teaching. Students and professors alike reported that network access was not always stable throughout the entire class and that this connection loss interfered with flow and concentration during classes^(19),(23),(32)-(35),(39)-(40),(45)-(46)^. Another factor is that, when establishing this type of education, lack of Internet access by everyone was not considered^(31)^, some students depended on the physical facilities of their colleges to study and, as they were closed during the pandemic period, they faced the financial difficulty of acquiring technological devices to attend classes, in addition to the difficult access to materials in libraries^(25),(29)^.

For nurses’ training, the acquisition of theoretical knowledge and practical skills is extremely important. Remote teaching was able to substitute face-to-face classes and meet the need for theoretical learning; however, the clinical practice was impaired^(29),(37),(39),(41)^. The studies showed that, in many cases, there was lack of face-to-face practice or sudden pauses due to new contamination waves and, with that, reduction of opportunities in the services^(23)^ and in the time of practical classes to avoid contagion, thus corroborating the difficulty of developing the clinical competence necessary for Nurses^(35)^.

Regarding this aspect, it is highlighted that, in Nursing, learning occurs through social interactions, which start from the first semesters of the course, considering that Nursing care is realized on human beings, that is, through the professional-patient relationship^(10)^.

Therefore, it is understood that the interactions promoted in face-to-face teaching are fundamental for learning, although they are not the only determinants for the occurrence of intellectual development, whether in the scope of Nursing or in any other context^(47)^.

Given this context, it becomes indispensable to think about strategies that minimize the negative repercussions of Nursing training through remote teaching, especially regarding the acquisition of skills and competencies, as a way of filling some gaps in terms of content and practices that were somehow not learned effectively.

As an intervention proposal to remedy possible gaps in practical training in Nursing, Problem-Based Learning emerges as an example of teaching modality, being established by the constructivist perspective that articulates action and experience with theoretical content, through reflection and problematization from experiences of the professional practice toward the search for possibilities of intervention on reality. Such a method is student-centered, developed in small groups and provides more pleasant, dynamic, solid, critical and understanding learning^(27)^.

Another suggestion is to carry out university extension courses for newly graduated nurses and also those with more years of training, but who realize the need for professional updating and training. In the courses, practical classes can be offered in the laboratory and/or in health services for the review of Nursing techniques and procedures as a way to improve the knowledge and skills inherent to the profession.

The contributions of this study to the Nursing and Health areas are based on the repercussions imposed by the pandemic on the training and practice of future nurses and recent graduates during the current health crisis. Studies like this, exploring the repercussions of the pandemic on Nursing education, are incentives to reflect on teaching methods and their repercussions on nurses’ training, in addition to instigating and enabling the development of fruitful teaching practices that favor learning of safe and good quality Nursing care.

This study has limitations related to the predominance of studies with low levels of evidence, as the methodologies come from reflections, experience reports and qualitative studies. Despite these limitations, this review highlights the methodological rigor required by the JBI and the mapping of diverse evidence on the pandemic repercussions on Nursing education.

## Conclusion

The current paper allowed mapping the diverse scientific evidence about the repercussions imposed by the pandemic on Nursing training. It is concluded that remote teaching was a timely emergency solution for the continuity of academic training; however, this educational modality exerted positive and negative repercussions that need to be rethought and considered for a better systematization of teaching-learning in other contexts similar to the COVID-19 pandemic.

Among the reflections raised, it is worth mentioning the interpretations of the negative impacts for the elaboration of strategies that minimize possible gaps in the training of recent graduates and future nurses who had, at some point in their academic path, emergency remote teaching as an educational modality.

Therefore, the importance of new research studies that evaluate students and nurses on the impacts of the pandemic on training and practice in Nursing is highlighted as a way of knowing the weaknesses of this type of teaching and providing a basis for the elaboration of public policies that encourage access to the Internet and to digital equipment for all students, fostering teachers’ training to perform in emergency remote teaching. It is also encouraged that researchers carry out experimental studies in order to demonstrate such repercussions in the clinical practice.
